# Primary Care Provider Views About Usefulness and Dissemination of a Web-Based Depression Treatment Information Decision Aid

**DOI:** 10.2196/jmir.5458

**Published:** 2016-06-08

**Authors:** Julie Beaulac, Robin Westmacott, John R Walker, Gohar Vardanyan, The Mobilizing Minds Research Group

**Affiliations:** ^1^ The Ottawa Hospital Psychology Department Ottawa, ON Canada; ^2^ The Ottawa Hospital Research Institute Ottawa, ON Canada; ^3^ The University of Manitoba Department of Clinical Health Psychology Winnipeg, MB Canada; ^4^ The University of Manitoba Winnipeg, MB Canada

**Keywords:** decision aid, depression, treatment, dissemination

## Abstract

**Background:**

Decisions related to mental health are often complex, problems often remain undetected and untreated, information unavailable or not used, and treatment decisions frequently not informed by best practice or patient preferences.

**Objective:**

The objective of this paper was to obtain the opinions of health professionals working in primary health care settings about a Web-based information decision aid (IDA) for patients concerning treatment options for depression and the dissemination of the resources in primary care settings.

**Methods:**

Participants were recruited from primary care clinics in Winnipeg and Ottawa, Canada, and included 48 family physicians, nurses, and primary care staff. The study design was a qualitative framework analytic approach of 5 focus groups. Focus groups were conducted during regular staff meetings, were digitally recorded, and transcripts created. Analysis involved a content and theme analysis.

**Results:**

Seven key themes emerged including the key role of the primary care provider, common questions about treatments, treatment barriers, sources of patient information, concern about quality and quantity of available information, positive opinions about the IDA, and disseminating the IDA. The most common questions mentioned were about medication and side effects and alternatives to medication. Patients have limited access to alternative treatment options owing to cost and availability.

**Conclusions:**

Practitioners evaluated the IDA positively. The resources were described as useful, supportive of providers’ messages, and accessible for patients. There was unanimous consensus that information needs to be available electronically through the Internet.

## Introduction

Informed decision-making is essential to good patient health outcomes. Patients are more likely to initiate and continue treatment when informed about choices [1], facilitating a more cost-effective use of health care resources [2]. Shared decision making is associated with improved quality of the decision in terms of knowledge and values and improvements in treatment progress [3]. For depression, higher participation is associated with improved treatment adherence and health outcomes [4-6].

One tool for facilitating informed decision making on health is the information decision aid (IDA), which engages patients in the decision-making process and helps them to make choices among different treatment options. Information decision aids present information about a condition, associated medical tests and treatment options, and the probabilities of risks and benefits of the different options to assist patients with the decision-making process [7]. Information decision aids can be used as self- or practitioner-administered tools, alone or as part of structured counseling or patient education [8]. The importance of IDAs as a tool to support decision making brings forward the question of their effective dissemination in primary health care settings.

Attitudes of clinicians toward Web-based decision aids and Web-based tools to guide the treatment process have been found to be favorable, including for depression [9] and other mental health conditions (eg, psychosis; [10]). However, challenges have been identified related to implementing tools in actual practice [9,10]. A recent review indicated that adoption of IDAs in clinical practice has been very limited [11], possibly because of clinician concern about the content of the decision aids and about how well the use of IDAs fits into the traditional workflow in primary care settings. The International Patient Decision Aid Standards (IPDAS) criteria [12] emphasize the importance of the involvement of clinicians in real-world settings in evaluating and planning for the implementation of IDAs [13], and more engagement of clinicians may improve implementation [14].

Primary care professionals have important potential in disseminating IDAs about many issues including mental health. There is a gap in knowledge related to dissemination of Web-based tools for mental health, particularly from the perspective of health providers. To effectively disseminate such tools, we need to understand where health professionals currently go for information, how they provide information to patients, and how resources can be made available to better assist them in providing care for common mental health problems such as depression.

There are few IDAs available in the mental health area [5,6]. The ones that are available tend to focus on medication treatment rather than the wider range of treatment options that are available to consumers [15]. Our team, consisting of mental health professionals and young adults interested in mental health, has developed an IDA for the Canadian public focused on treatment of depression. The development of the IDA was guided by the IPDAS criteria [12]. The developers did extensive work to explore the information needs and preferences of members of the public concerning treatment options for depression and anxiety [15,16]. Consumer preference research [15] indicates that some segments of the public are interested in information available on the Internet accessed independently, whereas others are interested in traditional paper format accessed in consultation with health professionals. The IDA provides information in either (1) Web format or (2) brochure format, which may be downloaded from the website by consumers or health care providers. The IDA addresses a wide range of topics judged to be important by consumers [16] with information presented in modules focused on particular consumer questions with up-to-date evidence and references. Many of the topics have not previously been addressed in available information on the Internet and in patient brochures. The IDA differs from many others in that it is intended for use at any point in the treatment process and may be accessed by interested parties independently or in consultation with a health professional—before, during, or after a health care encounter. It may be used when considering treatment options, when making changes to treatment, and when considering stopping treatment. The resources are available on the Internet in English and French [17] (see Multimedia Appendix 1), have a Creative Commons copyright, and may be saved and disseminated in either the Web version or in fact sheet (pdf) versions (see Multimedia Appendix 2). Consistent with the recommendation of the IPDAS criteria that frontline clinicians be involved in the evaluation and dissemination of the IDA, the purpose of this study was to obtain the opinions of primary care providers about: (1) current information resources for primary care patients concerning treatment options for depression; (2) their opinion about the Informed Choices Depression IDA as a resource for patients in primary care; and (3) facilitators and barriers for the dissemination of the resources in primary care settings.

## Methods

### Focus Groups

A total of 5 focus groups were conducted at 2 sites of the Ottawa Hospital Academic Family Health Team and 3 large primary care clinics operated by the regional health authority in Winnipeg. Winnipeg and Ottawa are medium-sized cities with populations in the range of 600,000 to 1,000,000 in Central Canada and Eastern Canada, respectively. Each of these primary care settings serves high-need patients including the elderly, children, pregnant mothers, and lower income residents. In the Canadian system, there is no charge to the patient for a physician visit, and there is some coverage of drug costs (depending on the province) but limited availability of counseling and psychotherapy by providers other than physicians. The primary care clinics where the focus groups were held have greater access to nurses and in-house counselors than most primary care settings in the country. Table 1 summarizes the clinicians participating in each group.

 

**Table 1:** Focus group participants.

Site	Family physicians	Nurses or nurse practitioners	Social workers or mental health workers^a^	Other	Years of practice^b^	Male or female
	N	N	N	N	M (Range)	N
Ottawa 1	2	4	1	1 resident	27.1 (14-39)	2/6
Ottawa 2	5			1 resident 1 pharm^c^	23.8 (12-35)	3/4
Winnipeg 1	7	3/2	1	1 resident	9 (1-35)	6/8
Winnipeg 2	2	2/1		1 OT^d^ 1 clerk	19.5 (1-41)	2/5
Winnipeg 3	5	3/1		1 manager 1 OT 1 PCA^e^	23.1 (7-40)	4/8

^a^Social workers and mental health workers had generally completed masters’ level training.

^b^Years of practice includes clinical staff only and not administrative staff.

^c^pharm: pharmacist.

^d^OT: occupational therapist.

^e^PCA: primary care assistant.

### Procedure

The focus groups ran for approximately 1 hour and were held at regular team meeting times when possible so as to engage as many practitioners as possible from each setting. They were facilitated by a psychologist experienced in leading focus groups (ie, JB for Ottawa clinics, JW for Winnipeg clinics). The facilitator ensured that all participants had an opportunity to speak. There was also a notetaker present for each focus group, and participants received a $25 gift card for their participation. Seven questions guided the focus group discussion. The first groups of questions targeted: (1) what questions do providers hear from patients about treatments for depression; (2) how do they handle these questions; and (3) how do they provide information to patients. After these introductory questions, focus group participants received an example fact sheet from the IDA, a list of the fact sheets in the overall IDA, and a business card suitable for use with patients to inform them about the Web address for the IDA. Participants were asked: (4) whether and how they might consider using the materials; (5) the best ways to make this information available to patients; (6) times when this material would be especially relevant; and (7) how might this material be disseminated to health care providers.

### Data Analysis

Focus groups were digitally recorded and transcripts created. Written facilitator notes taken during the focus groups were later filled in by reviewing the recordings. A qualitative framework analytic approach [18,19] was used. This approach is used for applied qualitative research that is both grounded and inductive in the accounts and observations of the people studied but starts deductively from clearly identified research questions and preset objectives. During the first stage of data analysis, each group's transcript was reviewed several times by 1 member of the research team (JB, JW, and RW), and preliminary themes were identified. The transcript was then reviewed on a line-by-line basis, and themes were coded. By the final group transcript, no additional themes were identified, and thematic saturation deemed reached. The coded transcripts were reviewed and compared by a second team member for accuracy and agreement on codes or categories and to search for missed concepts or themes. Disagreements were discussed and consensus achieved. Themes were summarized and interpreted in collaboration among the 3 team members. Participant background data were compiled and means computed.

## Results

### Focus Group Themes

#### The Key Role of the Primary Care Provider

An early theme was the key role of the provider, including developing and maintaining trust, responsibility for the overall health of the patient, assessment and diagnosis, reviewing treatment options, and normalizing the patient’s experience. One practitioner expressed this role clearly when saying, “the fundamental interaction between my patient and myself is predicated on trust…If you do not have that trust, it is very hard to do anything that leads to anything effective.” Practitioners expressed concerns about time pressure for tasks such as familiarizing themselves with patient resources, helping patients to access information, and discussing treatment options.

#### Questions About Treatments for Depression

Practitioners reported fielding many questions from patients. The most common are about side effects from medication (eg, sexual side effects, weight gain). Several providers said they take many additional questions about medications: “Most people have questions around effects of pharmacotherapy—Do I need it? What is the effect? How do you decide when I need it? How long will it take to work? How long do I have to be on it?” Other concerns are about becoming addicted, results to expect, when to stop, what happens when one stops, and comparing or changing medications.

Practitioners also described high patient interest in alternatives to medications. Patients ask questions about the effectiveness of counseling, light therapy, herbal remedies, exercise, and self-management including books and Web-based resources. Common questions mentioned specific to counseling included where to access services, provider recommendations for specific practitioners, cost, and how to access free or longer term therapy.

#### Treatment Barriers

A number of practitioners indicated that they direct patients to psychotherapy, mindfulness, healthy living (eg, exercise) and/or provide counseling themselves. Cost and limited access to resources are common barriers discussed, particularly related to nonpharmacological treatments. Other barriers include patient knowledge, limited social support, depressive symptoms, and concerns regarding stigma.

#### Sources of Information

It was clear that practitioners develop their own style of responding to patients, some relying mainly on discussion, whereas others using resources such as pamphlets, websites, or mobile phone apps. Main sources of information described included Web-based resources, crisis lines, community support groups, and written information. Some prefer to be familiar with resources before making recommendations. This is challenging because time to review information sources is limited.

The most commonly used written materials are local crisis line pamphlets and cards. Next were Web-based resources, especially for younger patients. Specific websites mentioned were the Canadian Mental Health Association, MoodGYM, AnxietyBC, and local websites (eg, hospital, self-help organization, or the clinic website). “I refer everyone to ementalhealth.ca,” indicated 1 provider. When using Web-based sources, some mentioned that they will show patients the source, print out the information, and review during the appointment and encourage the patient to return to discuss. For example, 1 provider said, “I will make myself a note to ask them if they went to the site or read the handout.”

Although practitioners described somewhat less frequent use of written information, in part because it is difficult to have the needed information available during the patient encounter, some practitioners indicated that they often used written information.

#### Concerns About Currently Available Information

Concerns were expressed regarding the quality of available information. Specific concerns related to reading level of materials, access to Canadian information, limited quality information on medication, and a general concern with the quality and validity of information were available on the Internet and in the media (eg, Dr Oz). Too much information was described by a number as not being helpful and sometimes causing alarm (eg, long lists of medication side effects), 1 practitioner stating, “too much information can actually dissuade them from getting well.”

#### Providers’ Opinions of Informed Choices IDA

Practitioners responded positively to Informed Choices Web-based IDA. The general opinion was that the resources seemed useful, supportive of providers’ messages, and accessible to patients in terms of language, format, and length: “It looks like it is easy to navigate—handouts are bullet points, not a big paragraph—not overwhelming.” The option of both handouts and Web-based formats was viewed positively in that the resource was perceived to appeal to different patient groups. Practitioners described the resources as relevant at any point in the treatment process. The importance of resources that patients can share with support persons was also highlighted: one practitioner described the IDA as “information to start conversations.”

Response to a sample of a business card with the website address and key information was particularly positive. Practitioners liked the color (bright blue), the graphic, and the convenient size. Consistent with views expressed regarding dissemination methods, some reported that they would use the cards, whereas others would not. One expressed liking the cards because patients “can look at what they were interested in versus what we think they are interested in.” A number thought that making the cards available in the waiting room would be convenient for patients and might initiate helpful discussions in the meeting with the practitioner.

In considering the range of topics available in the IDA, additional content areas were suggested such as materials on postpartum depression, pregnancy and breastfeeding, youth and depression, co-occurring disorders, substance use and depression, and links to local treatment resources.

#### Disseminating the IDA

There was consensus that information needs to be organized and available to practitioners electronically; captured by the following: “I am not blaming anybody; it is just too busy…We have to think of electronic solutions.” Practitioners expressed the need to give patients “access to a smorgasbord that is appropriate, edited, and maintained.” Some emphasized the need for information to be all in one place, easy to access, and for a coordinated clinic-wide dissemination strategy (eg, standardize practice to include in counseling session). One provider said, “I like having a central repository of a bunch of resources so that when I want to share resources, I can go to one place that will have multiple things so I do not have to search around.” Another stated “[we are] like jugglers with 10 problems with that patient…so the last thing on my mind is where is the damn card…I want to make sure the patient is not suicidal.” Accessibility suggestions included linking the resource to a shared computer network. The primary care settings varied in terms of Internet and printer access in clinical areas. Practitioners suggested a variety of approaches ranging from linking the IDA to the electronic medical record (EMR) system, having a link to the IDA website in bookmarks, or hosting the fact sheets on the clinic computer desktops. Managing patient resources is a significant challenge, given the broad range of problems managed in primary care.

When used, written information from the Web was viewed as helpful in reinforcing the provider’s message. The importance of tailoring information was highlighted, 1 describing use of written information when there is insufficient time “to really engage [patient] in the conversation you would like.” Posters on clinic bulletin boards were not reported as useful in general, although a few suggested resources could be kept in the waiting area; some mentioned printing handouts for patients to take home, particularly for patients without access to computers. “They need the information to go home with.”

Practitioners suggested a range of outlets for disseminating material on a wider scale, including the Internet, through professional associations, consumer-focused organizations, pharmacies, schools, employee assistance programs, insurance companies, and word of mouth.

## Discussion

### Principal Findings

Practitioners reported a need for quality information and resources for treatment of depression for both patients and providers. The findings support the use of multiple information channels (Web and print) to maximize dissemination of health information to clinicians and patients [15]. An increased preference (among both providers and patients) was reported for Web-based resources [20]. Nonetheless, the ongoing utility of written information was also stressed, and preference on source and format of information varied, consistent with research focused on patients [15]. Providers strongly voiced the need for treatment information to cover a broad range of questions about treatment options among varied patient populations at the same time as remaining concise. Many of the common questions identified by primary care providers related to topics covered in detail in the IDA. Although it is beyond the scope of an IDA to increase the availability of resources (a concern expressed by many primary care providers), the IDA has information for the person seeking treatment on identifying appropriate resources and managing barriers related to cost and availability of services. Many providers indicated that there are significant challenges in accessing and maintaining appropriate information for patients in the context of currently available resources. The need for tools to be accessible on the Internet and at low to no cost is supported by other research [9].

The specific Web-based IDA reviewed in this study was evaluated positively for use by providers in interactions with patients and for use directly by patients. Consistent with other IDA research [21], this IDA was viewed by clinicians as a helpful tool for communicating information between patients and providers. They found the range of topics that covered helpful and suggested several topic areas that would be important to develop as the materials evolve. They also provided specific suggestions for where to disseminate the IDA to reach the public. The response to the IDA seemed to be more positive than that described in a recent review [11] on IDAs in general. This may be because patients dealing with problems such as depression see themselves as taking a more active role in treatment and as having more treatment options than for many other health problems [16]. Furthermore, the IDA was designed for independent use by the consumer, so, it puts less demand on the health care provider’s time than some other IDA resources.

In considering dissemination in primary care, providers emphasized the importance of making the material easily available during patient encounters. It is clear that IDAs need to be at providers “fingertips” to get used. Given the expanding technological applications used within health, it will be important for future research to identify which apps (eg, Web, mobile phone technology) are most powerful for getting health information to both providers and consumers. A revision of the website (hosting system, not content) is currently underway so that the IDA may be conveniently viewed on the mobile phones that are increasingly used by young adults as a primary source of information. The use of business cards as a method to inform consumers about the availability of the IDA was strongly endorsed by primary care providers. On the clinician side, different settings have widely varying availability of electronic resources, and it will be important to develop approaches to dissemination that consider this reality and that integrate well with the systems that primary care providers use. Developing approaches to integrating this and other IDAs with common EMR systems will be important for successful implementation. It would be helpful in future research to evaluate the dissemination and implementation of the IDA in routine practice in a variety of primary care settings [11] and to consider the impact of the IDA on the experience of patients and providers.

### Strengths and Limitations of the Study

Although the study involved primary care providers in 2 regions of Canada serving varied patient populations, there are a number of limitations to consider. The clinics were large, serving urban areas, and tended to have staff members from several disciplines. The opinions of practitioners working in smaller clinics or serving small and remote communities may differ. Although it is possible that participants were influenced to report positive opinions on the IDA as a result of the involvement of one of the IDA developers in this study, this potential bias was mitigated by interviewing across 2 regions and having another researcher (JB) lead the study in one of the regions.

### Conclusions

The Informed Choices IDA [17] was viewed positively, and suggestions for dissemination were provided that are consistent with those of the literature. Namely, a mix of dissemination approaches with increasing importance place on electronic dissemination. These findings will be helpful in the planning of dissemination of treatment information for primary care settings.

## Appendix

Multimedia Appendix 1

Multimedia Appendix 2

## Abbreviations

EMR: electronic medical record
IDA: information decision aid
IPDAS: International Patient Decision Aid Standards
