# Supplementary material for: Primary Care Provider Views About Usefulness and Dissemination of a Web-Based Depression Treatment Information Decision Aid
Source: J Med Internet Res. 2016 Jun 8;18(6):e153. doi: 10.2196/jmir.5458 (PMC4917726; doi:10.2196/jmir.5458)
Supplement: Multimedia Appendix 1 [file jmir_v18i6e153_app1.pdf]

Appendix 1: Example Screenshots of Informed Choices About Depression Website

INFORMED CHOICES ABOUT

DEPRESSION

What is depression?

Getting help

Types of treatment

Special topics

Personal stories

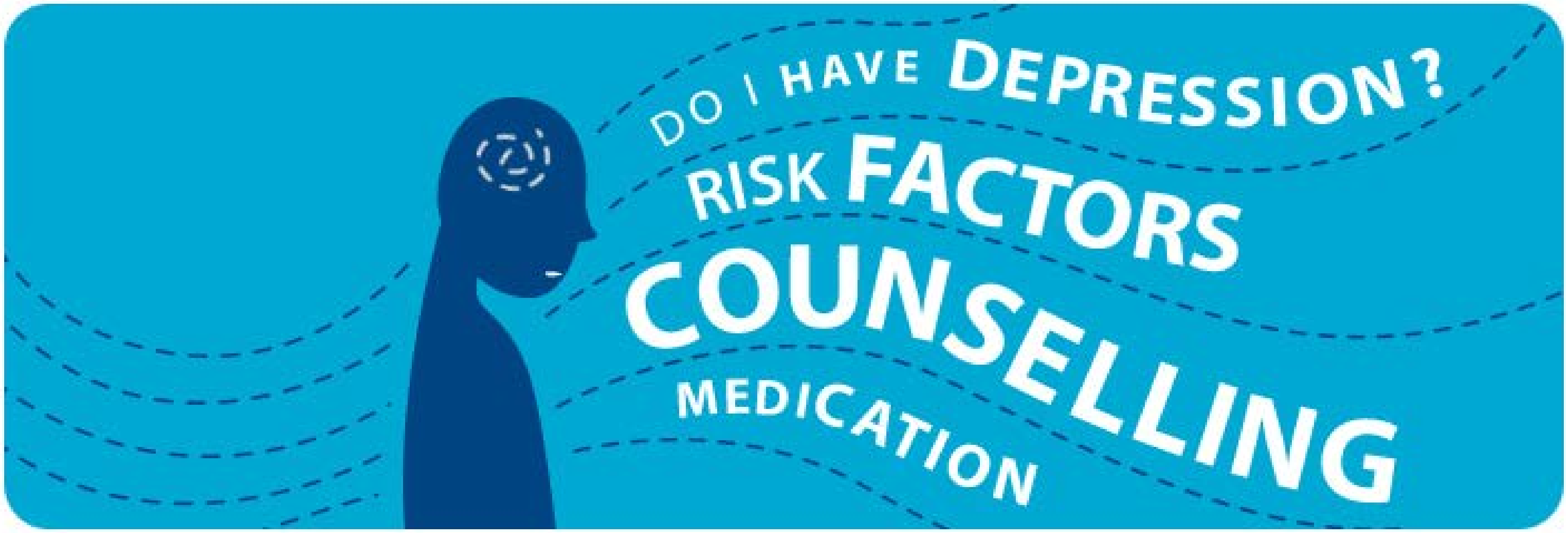

Are you looking for information about depression and treatments for depression?

This resource was developed by a team of researchers, professionals and young adults to answer the questions many people have about depression. It provides up-to-date information based on the best available research.

Do I have depression?

How do I get help for myself, a friend, or a family member?

What about self-help approaches?

Will counseling or therapy help?

Will medication help?

What about alternative treatments?

What is the cost of treatment?

Fact Sheets

These fact sheets are .pdf documents that may be saved, printed and shared, or posted on blogs and websites free of charge. They contain the same information as the website, but in some cases they are less detailed.

General Information Fact Sheets

Fact Sheet What is Depression 804.98 KB
Download

Fact Sheet Getting Help 554.66 KB
Download

Fact Sheet Helping a friend or family member 565.05 KB
Download

Fact Sheet Self Help Treatments 541.23 KB
Download

Counselling or Therapy Fact Sheets

Fact Sheet Counseling or Therapy for Depression 567.05 KB
Download

Fact Sheet What to expect during counseling 695.73 KB
Download

Fact Sheet Who Provides Counseling or Therapy 576.02 KB
Download

Fact Sheet Questions to ask when you are deciding on a therapist 593.38 KB
Download

Fact Sheet What happens when counseling is completed 567.30 KB
Download

Fact Sheet What are the risks of counseling 602.61 KB
Download

Fact Sheet How much does counseling or therapy cost 772.51 KB
Download

Fact Sheet Tips to managing the cost of therapy 649.28 KB
Download

Medication Treatment Fact Sheets

Fact Sheet Medication treatments for depression 815.39 KB
Download

Fact Sheet How long should you keep taking medication 803.21 KB
Download

Fact Sheet Medication Side Effects 828.86 KB
Download

Fact Sheet Reducing or stopping your medication 693.75 KB
Download

Fact Sheet Cost of Medication 577.53 KB

Please login to download

Fact Sheet Insurance Coverage for Medication Costs 572.48 KB

Download

Alternative Treatment Fact Sheets

Fact Sheet Dietary Supplements for Depression 523.99 KB

Download

Fact Sheet Exercise To Treat Depression 724.40 KB

Download

Fact Sheet Herbal Rx for Depression 515.70 KB

Download

Fact Sheet Light Therapy for Depression 545.15 KB

Download

Fact Sheet Mindfulness Meditation for Depression 505.48 KB

Download

Alternative treatments

Please see:

- Exercise to treat depression
- Dietary supplements to treat depression
- Herbal medicines to treat depression
- Light therapy to treat depression
- Mindfulness meditation to treat depression

For an infographic of alternative treatment information, click here
